# A social identity approach to COVID‐19 transmission in hospital settings

**DOI:** 10.1111/jasp.12948

**Published:** 2022-11-29

**Authors:** Niklas Hlubek, Anne Templeton, Kirsty Wiseman‐Gregg

**Affiliations:** ^1^ Department of Psychology, Old College The University of Edinburgh Edinburgh UK

## Abstract

The COVID‐19 pandemic poses a substantial risk of disease spread among healthcare workers (HCWs), making it important to understand what impacts perceived risk of COVID‐19 spread in hospital settings and what causes HCWs to mitigate COVID‐19 spread by following COVID‐19 safety measures. One determinant of risk perception and safe behaviors is the influence of seeing others as group members. The current study aims to (a) evaluate how social identification as an HCW and trust in co‐workers may influence perceived risk of COVID‐19 spread and (b) explore how communication transparency, trust in leaders, and identity leadership are associated with self‐reported adherence to COVID‐19 safety guidance. Using a correlational design, HCWs of a Scottish hospital were invited to participate in an online questionnaire measuring their perceptions of risk of COVID‐19 transmission, measures of social identification as an HCW, perception of leaders as members of the team, trust in co‐workers to follow the COVID‐19 guidelines and perception of leaders to manage COVID‐19 prevention effectively. Results showed that increased trust in co‐workers was associated with reduced risk perception of COVID‐19 transmission. Perceptions of transparent communication about COVID‐19 were found to be associated with increased adherence to COVID‐19 safety guidelines. Findings show the importance of the association between social identity processes and reduced risk perception and highlight the relationship between transparent communication strategies and self‐reported adherence to COVID‐19 guidelines, identity leadership, and trust in leaders to manage COVID‐19 appropriately.

## 1 INTRODUCTION

### 1.1 Background

The coronavirus disease (COVID‐19) global pandemic, which is caused by the novel severe acute respiratory syndrome coronavirus 2 (SARS‐CoV‐2), has to this date infected more than 511 million people worldwide (COVID‐19 Dashboard, April 2022). With the arrival of the Omicron variant, transmission risk has further increased, putting an additional strain on healthcare workers (HCWs) and hospitals settings (ARHAI Scotland, 2022; UK Health Security Agency, 2022). The urgency to protect HCWs has been stressed by the World Health Organization early over the course of the pandemic (WHO Geneva, 2020). It is important to better understand why transmission is happening in hospital settings to protect HCWs and ensure sufficient workforce capacity to tackle the pandemic.

Whereas earlier in the pandemic, uncertainty, and some conflicting advice about the dynamics of COVID‐19 transmission and its management led to confusion for HCWs and hospital managers, clear evidence now provides guidance on how to prevent COVID‐19 transmission (Bak et al., 2021). Next to personal protective equipment,  the implementation of physical distancing serves as a critical way to prevent the transmission of COVID‐19 in hospital settings and has repeatedly been demonstrated (Arora et al., 2020; Lewnard & Lo, 2020; Public Health England, 2022).

Research on healthcare‐associated outbreaks of COVID‐19 suggests that HCW‐to‐HCW transmission represented the likely source of outbreaks and insufficient physical distancing appeared to be one of the leading reasons for transmission (Jørstad et al., 2020; McMichael et al., 2020; Schneider et al., 2020; Schwierzeck et al., 2020). However, in their work environment, HCWs are around multiple people and face numerous challenges, which make physical distancing difficult, such as crowded administrative meetings and clinical workrooms, ward rounds, or signing out (Arora et al., 2020; Keller et al., 2021).

HCW‐to‐HCW transmission is an issue for COVID‐19 safety and although information about effective mitigation measures exists (e.g., wearing personal protective equipment and physical distancing, Public Health England, 2022), less is known about barriers and facilitators that HCWs face when trying to navigate guidance.

To support protective measures in hospital settings, Houghton et al. (2020) conducted a rapid qualitative review on general respiratory infectious diseases and summarized factors that influence whether HCWs follow adherence to infection prevention and control (IPC) guidelines. The authors explored the views and experiences of nurses, doctors, and other HCWs and identified several barriers and facilitators of guidance adherence. One of the main findings of the review was that HCWs reported that clear communication strategies and sharing new information were seen as vital for the successful implementation of IPC guidelines. HCWs generally felt unsure about how to adhere to guidelines that were lengthy and ambiguous and often felt overwhelmed because guidelines changed constantly. The level of support that HCWs perceived to receive from management influenced their responses to IPC guidelines. Many HCWs also highlighted the importance of workplace culture as an influence on whether IPC guidelines were followed or not. Workplaces, where all staff adhered to IPC guidelines, created a culture whereby HCWs had a sense of “pulling together” (Corley et al., 2010) whereas, in workplace cultures of complacency, HCWs were less likely to adhere to IPC guidelines (Zinatsa et al., 2018).

While substantial evidence shows how COVID‐19 is transmitted and which safety measures can prevent transmission, a better understanding of social psychological processes that drive COVID‐19 mitigation behavior is crucial to improve strategies and protect HCWS. Several topics from the social and behavioral sciences are relevant to pandemics, such as research on risk perception, social contexts, and leadership. The way that individuals perceive and respond to the threats of COVID‐19 can be influenced by group processes and their norms of behavior. Leaders play an important role in the coordination of preventive COVID‐19 measures and trust in leaders is especially important for adherence to COVID‐19 guidelines (Bavel et al., 2020).

This study aims to investigate two research questions. First, we investigate how social identification as an HCW and trust in co‐workers may influence perceived risk of COVID‐19 spread. Second, we explore the association between perceived communication transparency and self‐reported adherence to COVID‐19 guidance, and the role of trust in leaders and prototypical leadership for this association.

### 1.2 Social identity and the role of trust in risk perception

To investigate how social identification may be associated with trust in co‐workers and risk perception, it is important to investigate how psychological processes influence group processes. An established theoretical framework to understand group processes within organizational settings is the social identity approach (SIA, e.g., Reicher et al., 2010), which originated from social identity theory ([SIT]; Tajfel & Turner, 1979) and self‐categorization theory (SCT; Turner et al., 1987). The key principle of the SIT describes that the sense of self‐definition is not only derived from individual traits and qualities, but also from the groups that people categorize themselves as members of their social identity (Turner et al., 1987). In other words, social identity refers to the *internalized group membership* of an individual, defining the person's sense of ‘‘who they are’’ in a particular social context. This means that people self‐define themselves as “we” instead of “I” when they see themselves in terms of their social identity.

SIT describes the existence of social identities for every person (e.g., as a member of your professional group, such as an HCW), where the group identity is shaped by certain behaviors and beliefs that are normative or nonnormative. In hospitals, HCWs from various professions collaborate to provide services to patients and their identity as an HCW consists of a well‐constructed set of norms, values, motives, and experiences that define the professional role (Warren & Braithwaite, 2020).

The COVID‐19 pandemic requires individuals to go against behaviors that were previously normative (e.g., HCWs comforting each other), and maintaining physical distance from ingroup members represents a unique challenge because people feel safe around group members (e.g., Alnabulsi & Drury, 2014; Neville et al., 2021). Evidence suggests that social identification influences the desire of individuals for close physical proximity to ingroup members (Novelli et al., 2013) and ingroup relations have also been linked to attenuated core disgust toward ingroup members (Reicher et al., 2016). Although these studies were not conducted in hospital settings or the high‐risk context of COVID‐19, insights from social identity research are highly relevant for effective responses to the COVID‐19 pandemic (Bavel et al., 2020).

Building on social identity theory and self‐categorization theory, Cruwys et al. (2020) proposed a social identity model of risk‐taking (SIMORT), which states that potential threats from ingroup members will be perceived as less risky and inspire greater risk‐taking behavior, than potential threats from outgroup members. It is argued that, because ingroup members are typically trusted to a greater degree than outgroup members, this may systematically affect the degree to which ingroup members are seen as a potential threat as individuals tend to use shared group membership as a heuristic for ‘‘safety.’’ Across eight studies, including the context of COVID‐19, findings showed that shared group membership might reduce risk perception, primarily because ingroup members are trusted more than outgroup members (Cruwys, Greenaway, et al., 2021; Cruwys, Stevens, et al., 2021).

**Figure 1:**
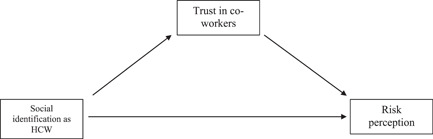
Association of social identification as an HCW and risk perception with meditator trust in group members. HCW, healthcare workers.

The current study builds on Cruwys, Greenaway, et al. (2021); Cruwys, Stevens, et al. (2021) model in the high‐risk context of a hospital setting to investigate the proposed mediating effect of trust in other HCWs to follow COVID‐19 guidance on the relationship between group identification as an HCW and risk perception of COVID‐19 co‐workers (see Figure 1).

**H1**. *Social identification as an HCW will be associated with a lower perceived risk of co‐workers transmitting COVID‐19, and this will be mediated by trust in co‐workers to follow COVID‐19 guidance*

### 1.3 The role of communication and leadership in adherence to safety guidance

#### 1.3.1 Communication transparency and seeing hospital leadership as part of the team

The second part of the study aims to explore the association between perceived communication transparency and self‐reported adherence to COVID‐19 guidance and the role of trust in leaders and prototypical leadership for this association. Even though there are detailed and available recommendations for COVID‐19 safety practices in hospital settings, guidance might not be easily transferrable because healthcare systems are highly variable in terms of their structure and workforce composition (ECDC, 2021; Pavolini & Kuhlmann, 2016). During a pandemic, HCWs experience rapid, unexpected change and to ensure that HCWs are informed about the latest safety measures it is crucial that information is transparently communicated (Spalluto et al., 2020).

Communication is essential in hospital settings and promotes workplace health and safety practices (Mascioli & Carrico, 2016). To improve collegiality and responsibility throughout staffing conditions and healthcare situations, internal communication should be structured, organized, and integrated (Foronda et al., 2016). Throughout the COVID‐19 pandemic, it is crucial that the communication between management and employees is timely and transparent when the latest safety‐related information is provided (Lanz et al., 2020; Spalluto et al., 2020).

Communication transparency is defined as the extent to which relevant information is shared amongst all stakeholders in the workplace (Haesevoets et al., 2021). In recent years, the empirical evidence on the value and effects of transparent communication in crisis situations has become more prevalent. Transparent communication has been shown to stimulate employees’ sensemaking and sense‐giving process during a crisis, highlighting the importance of communication strategies and participation (Kim et al., 2021). We hypothesize that in the context of COVID‐19 in hospital settings, higher perceived transparency of communication about safety guidance will be associated with higher levels of self‐reported adherence to the guidance.

Stranzl et al. (2021) investigated the role of transparent organizational communication as a resource for employees during COVID‐19 and found evidence to suggest that transparent communication was crucial to uphold job engagement throughout the crisis. Employees that did not perceive internal communication to be transparent were more likely to disconnect from their work roles. Moreover, Lee and Li (2021) tested a model in which transparent internal communication affects employees’ management of organizational changes in the context of the COVID‐19 pandemic. Results showed that transparent communication can impact how employees can cope with those changes and reduce their change‐related uncertainty.

The COVID‐19 pandemic creates the necessity for leaders to effectively coordinate and communicate to guarantee adherence to safety guidance. Especially in hospital settings, leaders have to ensure adherence to safety guidance and support HCWs by coordinating interactions of individuals and groups (Menon & Goh, 2005; Spalluto et al., 2020). Leaders that create and embed a sense of shared social identity (e.g., a sense of ‘‘we‐ness’’) and act as a prototypical member of the group can ensure that their guidance is followed (Abrams et al., 2021; Haslam et al., 2020).

Effective communication is a key attribute of successful leadership during crisis and providing clear and transparent information (e.g., explain why behavior is necessary; inform how to follow guidelines; provide regular updates) is an important aspect of legitimate leadership (Nicola et al., 2020; Templeton et al., 2020). Receiving clear information can enhance the perceived legitimacy of who provides the information and enhance the feeling of being part of the same group (Carter et al., 2013).

Although providing effective communication is key to successful leadership, research based on social identity theory and self‐categorization theory shows that another crucial element of successful leadership is being a *prototypical* leader. Being a successful prototypical leader involves acting in the same way the other group members are expected to act and acting in the group's interest (see Platow et al., 2015; Reicher et al., 2018; Steffens et al. (2013). In a hospital context, this would require staff in leadership roles to follow the COVID‐19 guidance they are asking their team to follow and acting in ways that support their team to follow the guidance. The closest representation of leadership in hospital settings are line managers, and therefore they provide the primary source of information about COVID‐19 safety guidance (e.g., through handovers or team meetings). Thus, leaders clearly communicating COVID‐19 safety measures to team members is one important part of leadership, but so is following the guidance themselves and helping the team to follow the guidance.

The current study aims to explore how the perception of leaders as prototypical members of the team influences the relationship between perceived communication transparency and self‐reported adherence to COVID‐19 safety guidance. As such, we aim to explore whether

**H2**. *perceiving leaders to be prototypical members of the team will mediate the association between perceived transparency of communication and self‐reported adherence to COVID‐19 safety guidance*.

#### 1.3.2 Trust in leaders to manage COVID‐19 appropriately

Trust among HCWs is a crucial element for effective healthcare delivery, yet less is known about the role of trust in leaders (Calnan & Rowe, 2008; Graham et al., 2015). Trusting relationships with leaders are needed so that HCWs are willing to seek information, cooperate in teams and communicate effectively (Marshall et al., 2013).

Effective communication strategies (e.g., clear and actionable information) have previously been linked to increased trust in authorities and compliance with guidance (Carter et al., 2013). In a study on leadership, trust in leaders and safety compliance in a hospital setting, results showed that leadership may not be directly effective in improving the safety compliance of subordinate nurses unless a leader first develops a trust‐based relationship with the subordinates. Among other factors, transparent communication strategies may be responsible for the subordinates’ trust in the leaders (Enwereuzor et al., 2020).

**Figure 2:**
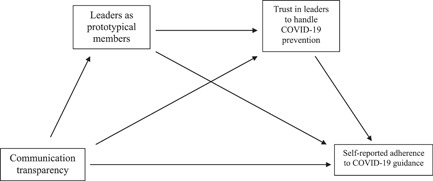
Proposed sequential mediation model

The current study aims to investigate how trust in leaders to handle COVID‐19 prevention appropriately might affect the relationship between perceived communication transparency and self‐reported adherence to COVID‐19 safety guidance in hospital settings. We hypothesize that the relationship between perceived transparent communication and self‐reported adherence to COVID‐19 guidance will be impacted by the extent to which HCWs believe that leaders can prevent COVID‐19 appropriately (see Figure 2).

We aim to explore whether

**H3**. *trust in leaders will mediate the association between perceived transparency of communication and self‐reported adherence to COVID‐19 safety guidance*.

#### 1.3.3 Sequential effect of prototypical leadership and trust in leaders

In addition to the hypothesized mediating effects of prototypical leadership and trust in leaders, both mediators might have a sequential effect on self‐reported adherence to COVID‐19 guidance. A large body of work has demonstrated that communication and cooperation among ingroup members is more effective and that members of the same group are trusted to a greater extent (Brewer, 2008; Greenaway et al., 2015; Tanis & Postmes, 2005). Leaders that are prototypical for the group tend to gain greater influence and be more trusted (Bavel et al., 2020; Haslam & Platow, 2001). The pivotal role of leaders for worker safety in healthcare settings has repeatedly been identified by researchers (e.g., Agnew & Flin, 2014) and evidence shows a relationship between trust in leaders and safety compliance in healthcare settings (Enwereuzor et al., 2020; Newman et al., 2014).

The current study also proposes a sequential mediating effect of both prototypical leadership and trust in leaders on the relationship between perceived communication transparency and self‐reported adherence to COVID‐19 safety guidance (see Figure 2). We included risk perception of COVID‐19 at the workplace as a control variable for the model to account for differences in HCWs perceived risk of transmission that may impact variables such as

We also aim to explore whether

**H4**. *the relationship between perceived transparency of communication and self‐reported adherence to COVID‐19 safety guidance will be mediated a sequential effect of perceiving leaders as prototypical members of the team and trust in leaders to mitigate COVID‐19 infections*.

### 1.4 Overview of the current study

Clear evidence exists on HCW‐to‐HCW transmission of COVID‐19 as well as physical and practical barriers in hospital settings, yet less is known about the social psychological processes that may impact self‐reported adherence to guidance. The SIA offers an established theoretical framework to explain the influence of group processes on the behavior of individuals and the role of leaders (Bavel et al., 2020).

The main objective of the current study is to promote the understanding of psychological processes that may cause barriers to following COVID‐19 guidance or facilitate adherence to guidance. This study will be divided to cover two research areas. The first part of the study will focus on the association between HCWs social identity and risk perception of COVID‐19 at the workplace, attempting to conceptually replicate findings from Cruwys, Greenaway, et al. (2021) and Cruwys, Stevens, et al. (2021) in a novel hospital context (*H1)*. The second part explores how the perceived communication transparency of HCWs is associated with self‐reported adherence to COVID‐19 guidance and specifically the role of leadership (*H2–H4)*.

## 2 METHODS

### 2.1 Ethics and open science

The study was approved by the host institution's ethics committee (reference 282‐2021/6). A full list of items used in this study and the scripts containing the described analysis can be found on the open science framework (https://osf.io/txua4/?view_only=417ce607354048c8b270d16f84dec9d4).

### 2.2 Procedure

All participants were HCWs at a hospital in Scotland and responded to a survey that was advertised via staff intranet, flyers, and snowball sampling by email between July 28 and August 13, 2021. HCWs were invited to participate in an online survey via the platform Qualtrics. After obtaining consent, participants were presented with items related to perceived communication transparency, followed by self‐reported adherence to COVID‐19 safety guidance, social identification as an HCW, trust that co‐workers were following COVID‐19 guidance, perceived risk of COVID‐19 transmission from co‐workers and perception that hospital leadership were prototypical members of the team. The survey took 5–10 min to complete. To ensure that participants paid attention when giving responses, an attention check was included halfway through the survey (‘‘This is an attention check. Please select ‘All of the time*’*”). No monetary reward was offered for participation, but participants did have the opportunity to leave their contact information in form of an email address to be included in a prize draw for multiple £50 e‐vouchers.

### 2.3 Participants

A total sample of 341 responses were collected from HCWs and 271 of those responses were complete. Participants were excluded if they did not give consent (*N* = 7) or failed to pass the attention check (*N* = 25). The total final sample included 239 participants.

### 2.4 Measures

#### 2.4.1 Social identification

This construct measured the four‐item measure of social identification  based on Postmes et al. (2013a, 2013b) adapted to the context of HCWs (e.g., ‘‘Being a Health Care Worker is an important part of how I see myself’’; *α* = .76) and evaluated on a 5‐point Likert scale (1 = *strongly disagree* to 5 = *strongly agree*), where high scores indicate high reported levels of social identification with fellow HCWs.

#### 2.4.2 Perceived communication transparency

This construct was measured using a 5‐item scale based on Haesevoets et al. (2021) and adapted to the context of COVID‐19 and HCWs (e.g., ‘‘I perceive the communication in my department about COVID‐19 to be clear’’; *α* = .89) and measured on a 5‐point Likert scale (1 = *strongly disagree* to 5 = *strongly agree*), where high scores indicate high reported levels of perceived transparent communication about COVID‐19.

#### 2.4.3 Risk perception when interacting with co‐workers

This construct was measured using a four‐item scale based on previous research on risk perception of COVID‐19 from Cruwys, Greenaway, et al. (2021); Cruwys, Stevens, et al. (2021) and Smith and Templeton (2022). Items were adapted to the context of COVID‐19 and HCWs (e.g., ‘‘I was concerned that my co‐workers could transmit COVID‐19’’; *α* = .82) and measured on a 5‐point Likert scale (1 = *strongly disagree* to 5 = *strongly agree* or respectively 1 = *very unsafe* to 5 = *very safe*), where high scores indicate high reported levels of perceived risk about COVID‐19 when interacting with fellow HCWs.

#### 2.4.4 Self‐reported adherence to guidance

This construct was measured using a four‐item scale based on Du and Liu (2020) and adapted to the context of COVID‐19 and HCWs (e.g., *‘‘*I incorporate the COVID‐19 safety measures into my work at all times’’; *α* = .93) and measured on a 5‐point Likert scale (1 = *strongly disagree* to 5 = *strongly agree*), where high scores indicate high self‐reported levels of adherence to COVID‐19 safety guidance.

#### 2.4.5 Trust in co‐workers to follow COVID‐19 guidance

This construct was measured using a one‐item measures based on previous research from Cruwys, Greenaway, et al. (2021); Cruwys, Stevens, et al. (2021). The item was adapted to the context of COVID‐19 and HCWs (‘‘Workers in my department can be trusted to follow the COVID‐19 guidance’’) and measured on a 5‐point Likert scale (1 = *strongly disagree* to 5 = *strongly agree*), where high scores indicate high reported levels of perceived trust in fellow HCWs related to COVID‐19 guidance.

#### 2.4.6 Trust in hospital leadership to manage COVID‐19 prevention

This construct was measured using a four‐item scale based on Carter et al. (2013) and adapted to the context of COVID‐19 and HCWs (e.g., ‘‘I trust that my line manager knows how to manage the COVID‐19 prevention appropriately’’; *α* =.96) and measured on a 5‐point Likert scale (1 *= strongly disagree* to 5 *= strongly agree*), where high scores indicate high reported levels trust in leaders to manage COVID‐19 safety measures.

#### 2.4.7 Prototypical leadership

This construct was measured using a four‐item scale based on previous research from Steffens et al. (2014) and adapted to the context of HCWs (e.g., ‘‘My line manager is a model member of the team’’; *α* = .96) and measured on a five‐point Likert scale (1= *strongly disagree* to 5 *= strongly agree*), where high scores indicate high reported levels of HCWs leadership as part of the team.

#### 2.4.8 Demographics

Demographic questions on gender, age group, and participants’ position at the hospital were included (see Table 1).

**Table 1:** Participant characteristic information (*N* = 239)

Demographics		Frequency	%
Gender			
Male		42	17.6
Female		195	81.6
Nonbinary		1	0.4
Did not disclose		1	0.4
Age			
18–24		6	2.5
25–34		52	21.7
35–44		67	28
45–54		112	46.9
55–64		2	0.8
Position in hospital			
Registered Nurse		90	37.6
Clinical support worker		12	5.3
Allied healthcare professional		34	14.2
Doctor		33	13.8
Nursing/Medical/AHP student		4	1.7
Administration		26	10.9
Nonclinical manager		3	1.3
Catering team		2	0.9
Other		35	14.6

### 2.5 Analytical strategy

The R programming language version 4.1.0 (R Core Team, 2021) was used for all analyses. Because items were adapted to the context of COVID‐19 and HCWs, an exploratory factor analysis using ordinary least squares with oblimin rotation was carried out using the psych package version 2.1.6 (Revelle, 2021). Descriptive statistics, including correlations between variables were obtained. The Lavaan package version 0.6‐9 (Rosseel, 2012) was used to perform a mediation analysis using structural equation modeling (SEM). Both models provided the regressions between each variable, the direct effects, the simple mediations via each mediator, and respectively the sequential mediation via both mediators in the second model.

In the first model, the independent variable (*X*) was self‐reported social identification as an HCW, the outcome variable (*Y*) was perceived risk of being infected with COVID‐19 due to a co‐worker, and the mediator (M) was trust in co‐workers to follow the COVID‐19 guidance. In the second model, the independent variable (*X*) was perceived communication transparency, the outcome variable (*Y*) was self‐reported adherence to COVID‐19 safety guidance, mediator 1 (M1) was perceived leadership as part of the team, and mediator 2 (M2) was perceived trust in leaders to manage COVID‐19 prevention appropriately.

A Sobel test was conducted to assess significant mediation effects using the bda package version 1.8 (Wang, 2021).

An alpha level of *α* = .05 was used for all statistical tests.

## 3 RESULTS

### 3.1 Descriptive statistics

Table 2 provides descriptive statistical information regarding the means, standard deviations, and correlations of all variables used in the analyses.

**Table 2:** Means, standard deviations (SD), and correlations of all variables

Variable	Mean	*SD*	2.	3.	4.	5.	6.	7.
1. Social identification	4.33	0.62	0.44**	−0.23*	0.21**	0.32**	0.28**	0.12
2. Trust in co‐workers	3.99	1.02		−0.44**	0.33**	0.36**	0.06**	0.19**
3. Risk perception	2.62	1.00			−0.17**	−0.25**	−0.35**	−0.04
4. Communication transparency	3.72	0.97				0.31**	0.45**	0.48**
5. Leadership as part of the team	3.48	1.19					0.73**	0.06
6. Trust in leaders	3.92	1.06						0.17**
7. Self‐reported adherence to guidance	4.57	0.79						

* *p* < .05;

** *p* < .001.

### 3.2 Factor analysis and descriptive statistics Model 1

#### 3.2.1 Factor analysis

To conduct an exploratory factor analysis for the constructs *social identification, trust in co‐workers, and risk perception when interacting with co‐workers*, the data were checked for their suitability for factor analysis. Linearity was checked through inspection of the linear and lowess lines for the pairwise relations of the variables and factorability was confirmed using a KMO test which yielded an overall *KMO* = 0.7 with no variable *KMO* < 0.50 as recommended by Field et al. (2012). Based on the examination of the scree plot and the results of a parallel analysis, the number of retained factors was established. The item factor loadings for social identification ranged from 0.49 to 0.73 and for risk perception when interacting with co‐workers from 0.58 to 0.91. The factor loading showed that trust in co‐workers did not map onto an own factor but cross‐loaded on social identification as an HCW (see Supporting Information: Table 1). However, trust in co‐workers was treated as its own factor throughout analyses due to its conceptual difference from social identification.

### 3.3 Regression analysis Model 1

SEM was used to test the mediating effect of trust in co‐workers on the association between self‐reported social identification and risk perception when interacting with co‐workers. The indirect effect of trust in co‐workers was calculated as the product of the effect of the relevant predictor on the mediator (social identification on trust in co‐workers) and the effect of the mediator on the outcome variable (trust in co‐workers on risk perception when interacting with co‐workers). We included self‐reported risk perception of COVID‐19 transmission as a control variable in the regression analyses.

The model was fitted using maximum likelihood estimation with robust (Huber–White) standard errors to account for the nonnormality of the standardized residuals. The full model showed satisfactory fit measures (*Χ* ^2^(15) = 222.982; root mean square error of approximation [*RMSEA*] = 0.049; complement factor I deficiency [*CFI*] = 0.986; trypsin‐like immunoreactivity [*TLI*] = 0.974; standardized root mean square residual [*SRMR*] = 0.041). Table 3 demonstrates the individual regressions between each of the variables in this analysis. The results are visualized in Figure 4. All regressions were statistically significant.

**Table 3:** Regressions for Model 1

	*β*	LCI	UCI	*SE*	*z*	*p*
TW–SI	1.140	0.447	1.833	0.354	3.226	.001
RP–SI	−.548	−1.009	−0.087	0.235	−2.330	.020
RP–TW	−.248	−0.415	−0.082	0.085	−2.924	.003

Abbreviations: *β*, standardized *β* coefficients; LCI, lower confidence interval; RP, risk perception when interacting with co‐workers; SE, standard error; SI, Social identification; TW, trust in co‐workers; UCI, upper confidence interval.

### 3.4 Mediation analysis Model 1

**Figure 3:**
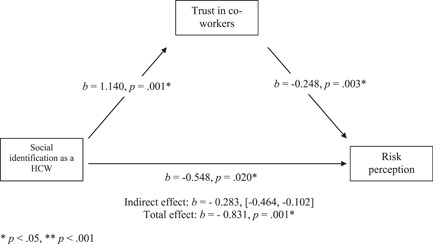
Simple mediation model for Model 1. *p < .05, **p < .001

The mediation analysis was conducted to investigate the hypothesis that the relationship between social identification and risk perception of COVID‐19 transmission will be mediated by trust in co‐workers (*H1*). Table 4 shows the total, direct and indirect effects for the mediation analysis. The results are visualized in Figure 3.

**Table 4:** Total, direct, and indirect effects for mediation analysis for Model 1

		*β*	LCI	UCI	*SE*	*z*	*p*
Total effect		−.831	−1.337	−0.325	0.258	−3.220	.001
Direct effect		−.548	−1.009	−0.087	0.235	−2.330	.020
Indirect effects							
SI–TW–RP		−.283	−0.464	−0.102	0.092	−3.070	

Abbreviations:  *β*, standardized *β* coefficients; LCI, lower confidence interval; RP, risk perception; SE, standard error; SI, social identification; TW, trust in co‐workers; UCI, upper confidence interval.

#### 3.4.1 Direct effects

There was a significant direct effect of social identification as an HCW on perceived risk that co‐workers would transmit COVID‐19 (*β* = −.548, *p* = .020, *z* = −2.330). Specifically, increased perception of social identity with co‐workers was significantly associated with reduced perception that co‐workers posed a risk of spreading COVID‐19.

#### 3.4.2 Partial mediation via trust in co‐workers

In line with H1, there was a significant indirect effect of social identification as an HCW on perceived risk that co‐workers would transmit COVID‐19 via trust in co‐workers (*b* = −0.283 [−0.464, −0.102]). Identifying as a member of the group of HCW was associated with lower perceptions that co‐workers could transmit COVID‐19, and this was mediated by increased trust that co‐workers would follow the COVID‐19 guidance. This mediation was also significant when evaluated in a Sobel test (*z* = −0.279, *p* = .005).

### 3.5 Factor analysis and descriptive statistics Model 2

#### 3.5.1 Factor analysis

To conduct an exploratory factor analysis for the constructs *communication transparency, perception of leaders as prototypical members of the team, trust in leaders, and self‐reported adherence to safety guidance*, the data were checked for their suitability for factor analysis. Linearity was checked through inspection of the linear and lowess lines for the pairwise relations of the variables and factorability was confirmed using a KMO test which yielded an overall *KMO* = 0.9 with no variable *KMO* < 0.50 as recommended by Field et al. (2012). Based on the examination of the scree plot and the results of a parallel analysis, the number of retained factors was established. The item factor loadings for communication transparency ranged from 0.66 to 0.87, for perception of leaders as prototypical leaders of the team from 0.75 to 0.95, for trust in leaders from 0.66 to 0.98, and for self‐reported adherence to safety guidance from 0.85 to 0.94.

### 3.6 Regression analysis Model 2

SEM was used to test for mediating effects of leadership as part of the team (M1) and trust in leaders (M2) on the association between perceived communication transparency and self‐reported adherence to COVID‐19 guidance. The indirect effects of the mediators M1 and M2 were calculated as the product of the effect of the relevant predictor on the mediator (communication transparency on M1 or M2) and the effect of the mediator on the outcome variable (M1 or M2 on self‐reported adherence to guidance). Risk perception when interacting with co‐workers was included as a covariate for the model.

The model was fitted using maximum likelihood estimation with robust (Huber–White) standard errors to account for the non‐normality of the standardized residuals. The full model showed satisfactory fit measures (*X* ^2^(155) = 242.102; *RMSEA* = 0.048; *CFI* = 0.979; *TLI* = 0.974; *SRMR* = 0.061). Table 5 shows the individual regressions between each of the variables in this analysis.

**Table 5:** Regressions for Model 2

	*β*	LCI	UCI	*SE*	*z*	*p*
PL–CT	.405	0.239	0.751	0.085	4.779	<.001
PL–RP	−.174	−0.308	−0.041	0.068	−2.555	.011
TL–PL	.561	0.456	0.666	0.054	10.470	<.001
TL–CT	.297	0.175	0.419	0.062	4.759	<.001
TL–RP	−.148	−0.233	−0.062	0.044	−3.375	.001
AG–CT	.508	0.330	0.626	0.091	5.593	<.001
AG–PL	−.085	−0.217	0.048	0.069	−1.254	.210
AG–TL	−.046	−0.231	0.139	0.094	−0.491	.624
AG–RP	.024	−0.058	0.107	0.042	0.576	.565

Abbreviations: AG, self‐reported adherence to guidance; *β*, standardized *β* coefficients; CT, communication transparency; LCI, lower confidence interval; PL, prototypical leadership; RP, risk perception when interacting with co‐workers; SE, standard error; TL, trust in leaders; UCI, upper confidence interval.

Risk perception when interacting with co‐workers was added as a covariate to all regression analyses. Increased perception that co‐workers could transmit COVID‐19 was significantly associated with reduced trust in leaders (*β* = −0.148, *p* = .001, *z* =  −3.375). Increased risk perception was also significantly associated with reduced perceptions of leadership as part of the team (*β* = −0.174, *p* = .011, *z* = −2.555). No association between risk perception and self‐reported adherence to COVID‐19 guidance was observed (*β* = 0.024, *p* = .565, *z* = 0.576).

#### 3.6.1 Mediation analysis Model 2

**Figure 4:**
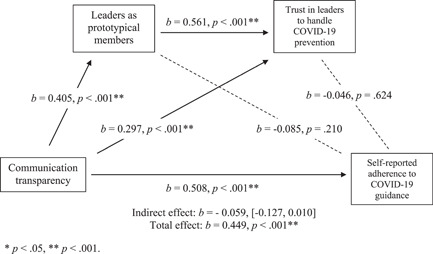
Sequential mediation model for Model 2. * *p* < .05, ** *p* < .001.

The mediation model evaluated whether perceived communication transparency would increase self‐reported adherence to guidance via two sequential mediators whereby leadership as part of the team was entered as the first mediator and trust in leaders as the second. The total, direct, and indirect effects can be found in Table 6. The results are visualized in Figure 4.

**Table 6:** Total, direct, and indirect effects for mediation analysis for Model 2

		*β*	LCI	UCI	*SE*	*z*	*p*
Total effect		.449	0.289	0.609	0.082	5.496	<.001
Direct effect		.508	0.330	0.626	0.091	5.593	<.001
Indirect effects							
Total indirect effect		−.059	−0.127	0.010	0.035	−1.675	.094
CT–PL–AG		−.034	−0.089	0.021	0.028	−1.226	
CT–TL–AG		−.014	−0.069	0.041	0.028	−0.490	
CT–PL–TL–AG		−.011	−0.052	0.010	0.021	−0.496	

Abbreviations:  AG, self‐reported adherence to COVID‐19 guidance; *β*, standardized beta coefficients; CT, communication transparency; LCI, lower confidence interval; PL, prototypical leadership; SE, standard error; TL, trust in leaders; UCI, upper confidence interval.

#### 3.6.2 Direct effects

There was a significant direct effect of perceived communication transparency on self‐reported adherence to guidance (*β* = 0.508, *p* < .001, *z* = 5.593). Specifically, increased perception of communication transparency was significantly associated with increased self‐reported adherence to guidance.

#### 3.6.3 Mediation via prototypical leadership and trust in leaders

Contrary to *H2*–*H4*, there was no indirect effect of perceived communication transparency on self‐reported adherence to COVID‐19 guidance via perceiving leaders prototypical members of the team (*β* = −0.034 [−0.089, 0.021]; *H2*), trust in hospital leaders (*β* = −0.014 [−0.069, 0.041]; *H3*) or a sequential mediation of both factors (*β* = −0.011 [−0.052, 0.031]; *H4*). However, there was a significant association between prototypical leadership and trust in leaders to manage COVID‐19 prevention (*β* = 0.508, *p* < .001, *z* = 5.593).

## 4 DISCUSSION

Outbreaks of COVID‐19 in hospital settings and infections of HCWs frequently originate from contacts with colleagues (Schneider et al., 2020). Appropriate safety measures (e.g., physical distancing) are essential to prevent transmission among staff members and social processes might play an important role in safety behavior. The current aimed to (a) evaluate how social identification as an HCW and trust in co‐workers may influence perceived risk of COVID‐19 spread and (b) explore how communication transparency, trust in leaders, and identity leadership are associated with self‐reported adherence to COVID‐19 safety guidance.

### 4.1 Overview of the current findings

Results of the first part of the study show that hospital staff members who identified more strongly as HCWs were more likely to trust their co‐workers, and thus, reported lower levels of perceived risk that co‐workers could transmit COVID‐19. The results confirmed our H1, showing that the association between social identification as an HCW and perceived risk of co‐workers transmitting COVID‐19 is mediated by trust in co‐workers to follow COVID‐19 guidance. These findings replicate the SIMORT of Cruwys, Greenaway, et al. (2021); Cruwys, Stevens, et al. (2021) in the novel context of hospital settings. Results indicate that even in a COVID‐19 context where the risk of disease spread is especially high, risk perception can be reduced.

The second part of the current study did not find evidence for Hypotheses 2–4, showing no mediating effect of perceiving leaders as part of the team or trust in leaders on the association between perceived communication transparency and self‐reported adherence to COVID‐19 guidance. However, even though none of the explored mediation effects were significant, the results give strong evidence for the direct effect of perceived communication transparency on self‐reported adherence to COVID‐19 safety guidance. This is in line with other research, highlighting the importance of transparent communication about COVID‐19 in hospital settings (Lanz et al., 2020; Spalluto et al., 2020). In particular, the results of the direct paths of Model 2 also show the important relationship between communication strategies, trust in leadership to handle COVID‐19 prevention effectively and seeing leaders as prototypical members of the team. Findings on the association between leadership as part of the team and trust in leaders are in line with previous literature on identity leadership, where higher levels of reported leadership prototypicality predict higher trust in leaders (Haslam et al., 2020; Krug et al., 2021)

### 4.2 Theoretical implications

Findings from the current study addresses the understanding of group processes, specifically trust in group members, which is important to model safety behavior related to the transmission of COVID‐19 in hospital settings. Trust in co‐workers is important for efficient delivery of healthcare (e.g., Marshall et al., 2013) and a facilitator for cooperation and solidarity in the context of COVID‐19 (Jetten et al., 2020). However, trust in co‐workers may pose an unnoticed risk for HCWs as it might reduce their risk perception of COVID‐19 spread and hence make HCW‐to‐HCW transmission more likely. These findings illustrate how a better understanding of group processes can be crucial for researchers and public health policy to predict and manage COVID‐19‐related risk behavior. Ingroup relations can attenuate perceived risk of COVID‐19, even in high‐risk contexts such as hospitals.

Findings on the association between perceived communication transparency and self‐reported adherence to COVID‐19 safety guidance confirm recent qualitative evidence on the importance of clear communication about safety guidelines in hospital settings. Houghton et al. (2020) found that constantly changing information and guidelines are challenging for HCWs and difficult for healthcare organizations to disseminate. HCWs valued clear strategies where any updates or changes are communicated in a timely manner and through multiple platforms or methods (e.g., posters, daily case conferences, summary notice at changeover). The current study additionally shows that the provision of clear communication is associated with increased perception of leaders as prototypical members of the team and trust in their ability to effectively manage COVID‐19 prevention.

Overall findings highlight the importance of social identity theory and self‐categorization theory in understanding how social processes may impact individual's responses to crisis situations like the COVID‐19 pandemic. While recent literature emphasizes the relevance of social influence and collective behavior in response to the COVID‐19 pandemic (see Jetten et al., 2020), to our knowledge, this is the first study that applies an SIA to the context of hospitals and HCWs. The current study tests previous models by focusing on exceptionally high‐risk environments where there is evidence of disease spread and knowledge of (un)safe behaviors. A better understanding of group processes related to adherence to guidance can help to mitigate the risk of COVID‐19 transmission, which is crucial for hospital settings to facilitate safety for HCWs and maintain a sufficient workforce capacity.

### 4.3 Practical implications

There are several implications of the current findings that can be used to guide the practical management of COVID‐19 transmission in high‐risk workplace environments such as hospitals. First, it is important for public health messaging to emphasize that people we are close to (e.g., co‐workers, friends) pose an important risk factor for the transmission of COVID‐19. Risk perception might be lower in networks where HCWs feel part of the same group, so even though hospital staff are aware of COVID‐19 safety guidance, they may be less likely to see co‐workers as a source of risk. Therefore, it is important to raise HCW's awareness of COVID‐19 spread among groups of colleagues. Furthermore, it is crucial to foster information sharing in hospital settings to provide HCWs with guidance on how gatherings with colleagues can be made safer.

Second, findings on the impact of trust on co‐workers are in line with qualitative insights of Keller et al. (2021) who found that familiarity and comfort with co‐workers, as well as the desire to maintain close relationships with fellow HCWs impacted physical distancing negatively. Multiple studies have described the negative impact of physical distancing on psychological well‐being and relationships, especially for HCWs (Gemine et al., 2021; Nguyen et al., 2020). Thus, it is highly important to support work relationships while maintaining physical distancing. Findings from the current study show that identification as an HCW was associated with trust that others would follow COVID‐19 guidance. This suggests that following the guidance is part of the group norms and could be harnessed to facilitate safety behavior.

Insights from research on the SIA could be highly important for organizational settings in response to crises situations like the COVID‐19 pandemic. Previous research demonstrated the importance of organizational approaches that stress the significance of collaborative teamwork as a key strategy for successful response to crisis. Porter et al. (2021) describe a ‘‘one‐team approach’’ to crisis management in a hospital setting in response to COVID‐19 that draws on the SIA. The Cleveland Clinic in Ohio (USA) emphasized collaborative teamwork on an organizational level and specifically focused on redeployment of workforce, engagement of key stakeholders and communication strategies. Positive relations between staff and leadership may be fostered through transparent communication approaches and leaders acting as prototypical group members.

Fostering collaboration, support, and cooperation in organizational settings may help setting up desirable organizational norms and values. Workplaces that do not foster these aspects of organizational culture have been linked to lower levels of organizational citizenship behaviors toward the organization and its members (Koc et al., 2021). Accordingly, organizations such as hospitals should aim to foster cultures where safety behaviors are established as values and goals to improve safety and strengthen organizational citizenship behavior.

### 4.4 Limitations and future directions

Some limitations must be considered when evaluating the current study. Participants were recruited from one hospital in Scotland during a period when community prevalence of COVID‐19 had temporarily decreased, and vaccination was already available. Although, most HCWs had been vaccinated long before survey responses were collected (July and August 2021). By March 2021, 95.7% of health and social staff in Scotland had received their first dose, 1.5% had booked an appointment to be vaccinated, and only 2.2% had decided not to be vaccinated (2.1% of HCWs staff said they had not been offered the vaccine; Public Health Scotland, 2022). We therefore do not suspect that social identification as an HCW was solely based on vaccinations. Future studies could investigate the relationship between risk perceptions of COVID‐19 and the beliefs about the COVID‐19 vaccine.

Previous research suggested that physical barriers (e.g., insufficient space, scheduled huddles) were reasons for nonadherence to COVID‐19 guidance (Houghton et al., 2020; Keller et al., 2021). The current study did not assess these additional factors that may have caused the nonsignificant relationship between trust in leadership and self‐reported adherence.

It is important to highlight that the present results are based on a cross‐sectional correlational design, hence, no causation can be inferred. The observed relationships give indication for the strength of the association between variables and should be interpreted with respective caution and it is important to note that the relationships between variables could reverse (e.g., trust in leaders could be placed in the model before prototypical leadership). Current results highlight the importance of communication transparency for adherence to guidance, and future research could investigate possible mediating effects of this variable on other relationships.

Some variables showed high means and relatively low standard deviations which could indicate possible ceiling effects and limit the current findings. In Model 2, responses for self‐reported adherence to COVID‐19 guidance might have been affected by a response bias of HCWs overestimating their adherence to guidance. Future research should include behavioral data to accurately measure adherence to COVID‐19 guidance.

Findings of the current study are part of an efficient response to the ongoing COVID‐19 pandemic and could have important implications for hospital context as well as other high‐risk work environments, including primary care facilities and community‐based facilities such as care homes. Research on the reasons for adherence to safety guidelines in the context of COVID‐19 is urgently needed, especially to help develop and evaluate interventions that create a climate of safety.

## 5 CONCLUSION

The present study shows new developments in understanding the underlying processes of perceived risk of COVID‐19 transmission and self‐reported adherence to COVID‐19 guidance. Results demonstrate that trust in co‐workers to follow the COVID‐19 safety guidance may mediate the relationship between social identification as an HCW and risk perception of co‐workers spreading COVID‐19 in a hospital setting. Furthermore, it highlights the importance of communication transparency on the relationship to adherence to COVID‐19 safety guidance and suggests that higher perceptions of transparent communication may be associated with viewing leadership as part of the team, and trusting leaders to know how to handle COVID‐19 prevention. The results replicate prior findings on the mediating effect of trust in members of the same group for risk perception (Cruwys, Greenaway, et al., 2021; Cruwys, Stevens, et al., 2021) and demonstrate that the SIA and its empirical evidence can be applied to the novel context of a hospital setting.

The reduction in perceived risk of COVID‐19 transmission due to social identity processes emphasizes the relevance and ability to utilize this theoretical approach when investigating health‐related behavioral processes. Future research should aim to evaluate the behavioral impacts of reduced risk perception and increased social identity processes for high‐risk work environments during COVID‐19. Evidence on the important association between leadership providing clear, actionable, and timely communication of COVID‐19 guidance and self‐reported adherence to the guidance should be used to further investigate how HCWs can be supported in their daily work life. With the ongoing pandemic, it remains crucial to support HCWs by understanding the social psychological facilitators and barriers they face when navigating the COVID‐19 guidance and teamwork in high‐risk environments.

## CONFLICT OF INTEREST

The authors declare no conflict of interest.

## Supporting information

Supporting information.

## Data Availability

The data that support the findings of this study are openly available in Open Science Framework at https://osf.io/txua4.
