# Supplementary material for: A social identity approach to COVID‐19 transmission in hospital settings
Source: J Appl Soc Psychol. 2022 Nov 29:10.1111/jasp.12948. Online ahead of print. doi: 10.1111/jasp.12948 (PMC9878075; doi:10.1111/jasp.12948)
Supplement: Supplementary file 1 — Supporting information. [file JASP-9999-0-s001.docx]

**Supplementary material**

**Table 1. Factor loadings for items in model 1**

|  | *Construct* |  | *Item* | *MR1* | *MR2* |
| --- | --- | --- | --- | --- | --- |
|  | Risk perception |  | “I was concerned that my co-workers could transmit COVID-19” | 0.869 |  |
|  |  |  | “I felt that my co-workers could put me at risk of being infected with the COVID-19 virus” | 0.845 |  |
|  |  |  | “How safe or unsafe do you feel about COVID-19 when interacting with your co-workers?” | 0.632 |  |
|  | Trust in co-workers |  | “Workers in my department can be trusted to follow the COVID-19 guidance” |  | 0.363 |
|  | Social identification |  | “I am proud to be a Health Care Worker” |  | 0.795 |
|  |  |  | “Being a Health Care Worker is an important part of how I see myself” |  | 0.754 |
|  |  |  | “I feel part of a team with my colleagues” |  | 0.493 |
|  |  |  | “I identify with my colleagues” |  | 0.432 |

**Table 2. Factor loadings for items in model 2**

| *Construct* | *Item* | *MR4* | *MR1* | *MR3* | *MR2* |
| --- | --- | --- | --- | --- | --- |
| Prototypical leadership | “My line manager creates structures that are useful the team members” | 0.95 |  |  |  |
|  | “My line manager creates a sense of cohesion within the team” | 0.948 |  |  |  |
|  | “My line manager acts as a champion for the team” | 0.945 |  |  |  |
|  | “My line manager is a model member of the team” | 0.751 |  |  |  |
| Trust in leaders | “I trust that my line manager knows how to manage the COVID-19 prevention appropriately” |  | 0.982 |  |  |
|  | “I have faith in my line manager to handle the COVID-19 prevention” |  | 0.976 |  |  |
|  | “I feel confident that my line manager is taking appropriate actions to successfully manage COVID-19 prevention” |  | 0.865 |  |  |
|  | “I believe what my line manager tells me about the COVID-19 prevention” |  | 0.663 |  |  |
| Communication transparency | “I think the information regarding COVID-19 is shared among all workers” |  |  | 0.872 |  |
|  | “I perceive the communication of COVID-19 safety information between the hospital and its workers to be clear” |  |  | 0.862 |  |
|  | “I think that hospital workers share information about COVID-19 safety guidance with each other” |  |  | 0.749 |  |
|  | “I think that hospital workers communicate honestly with each other about the COVID-19 safety guidance” |  |  | 0.718 |  |
|  | “I perceive the communication in my department about COVID-19 to be clear” |  |  | 0.659 |  |
| Self-reported adherence to COVID-19 safety guidance | “I abide by rules to prevent the spread of COVID-19 in my work” |  |  |  | 0.939 |
|  | “I wear the necessary personal protective equipment as required” |  |  |  | 0.869 |
|  | “I actively cooperate with the line managers to prevent the spread of COVID-19” |  |  |  | 0.857 |
|  | “I incorporate the COVID-19 safety measures into my work at all times” |  |  |  | 0.845 |
